# Acetylcholinesterase histochemistry (AChE) - A helpful technique in the diagnosis and in aiding the operative procedures of Hirschsprung disease

**DOI:** 10.1186/s13000-015-0443-5

**Published:** 2015-12-02

**Authors:** R. K. Agrawal, Nandita Kakkar, R. K. Vasishta, Vandana Kumari, R. Samujh, K. L. N. Rao

**Affiliations:** Department of Pathology, Post Graduate Institute of Medical Education and Research (PGIMER), Chandigarh, 160012 India; Department of Histopathology, Post Graduate Institute of Medical Education and Research (PGIMER), Chandigarh, 160012 India; Department of Pediatric Surgery, Post Graduate Institute of Medical Education and Research (PGIMER), Chandigarh, 160012 India

**Keywords:** Hirschsprung’s disease, Frozen section, Acetylcholinesterase (AChE) histochemistry, Ancillary technique, Diagnosis, Operative procedures

## Abstract

**Background:**

Hirschsprung’s disease (HD) is an anomaly characterized by the absence of myenteric and submucosal ganglion cells (GC) in the distal alimentary tract. Diagnosis of HD is made by the absence of GC and missing out on even a single ganglion cell can be very devastating. Acetylcholinesterase (AChE) histochemistry, done on frozen sections is said to be a very useful ancillary technique in the diagnosis and in aiding the operative procedures of HD.

**Methods:**

To assess this, 73 samples from 42 suspected/known cases of HD were subjected to frozen section analysis with rapid haematoxylin and eosin, toluidin blue stain along with AChE histochemistry. The remnant sample was paraffin embedded for routine haematoxylin and eosin staining.

**Results:**

On frozen section analysis, 33 samples showed absence of ganglion cells, AChE histochemistry showed a positive staining pattern in 17 samples and paraffin embedded routine, H&E stained sections showed absence of ganglion cells in 19 samples. Sensitivity and specificity of both tests ie frozen section rapid H&E/AChE histochemistry in the diagnosis of HD, were calculated taking paraffin embedded H&E stained sections as the gold standard. Sensitivity of frozen section rapid H&E in the diagnosis of HD is 57.57 % and specificity is 79.10 %. The p-value is <0.0001, which is significant. The sensitivity of AChE histochemistry in the diagnosis of HD is 90.47 % and specificity is 96.36 %. The p-value is <0.0001, which is significant.

**Conclusions:**

Acetylcholineesterase (AChE) histochemistry is a very useful ancillary technique in the diagnosis and in aiding the operative procedures of HD. It acts as a double check in the diagnosis of HD.

## Background

Hirschsprung’s disease (HD) [1–4] or congenital aganglionic megacolon was first described more than 100 years ago by Harold Hirschsprung. It is an anomaly characterized by the absence of myenteric and submucosal ganglion cells (GC), most commonly affecting the rectosigmoid region and is the most common cause of neonatal intestinal obstruction. It is a heterogeneous group of disorders in which abnormalities of the enteric nervous system are usually manifested as delayed (more than 48 h) passage of meconium in the newborn or chronic constipation in infants and children, frequently accompanied by abdominal distension and vomiting. Despite spectacular advances in understanding the genetic basis and pathogenesis of HD in the last few years, the ability to identify this condition continues to depend mostly on a skillful pathologic analysis and the use of routine hematoxylin and eosin (H&E) stains. The increasing use of the less cumbersome and patient friendly suction rectal biopsy by the pediatric surgeons has turned out to be a more error prone procedure for the pathologists, as it can be a difficult task to look for the GC in the sub-mucosal plexus, since they are fewer in number, can be singly placed and randomly distributed. More so, the diagnosis of HD is made on the absence of GC and missing out on even a single ganglion cell, can be very devastating and can totally change the diagnosis. Recently there has been an increasing trend at some centers to perform the single stage transanal endo-rectal pull through (TEPT) in selected cases. This has further increased the pathologists’ responsibility, as a frozen section diagnosis to confirm the level of the ganglionic segment is mandatory, before the colo-anal anastomosis is performed. AChE histochemistry is being performed in various centers worldwide, as an ancillary technique in the diagnosis and aiding in the operative procedures of HD. The histochemical diagnosis of HD is based on the fact that the cholinergic nerve fibers of the aganglionic segment are prominent and that these fibers contain an increased amount of AChE [5], which shows a positive staining pattern with AChE histochemistry. So, the aim of this study was to assess the accuracy of rapid H&E stain and AChE histochemistry in the diagnosis and in aiding the operative procedures of HD.

## Methods

All routine diagnostic surgical rectal biopsies (full thickness) done on suspected cases of HD, doughnuts derived at the time of colostomy closures (Duhamel surgery) and biopsies from one stage transanal endorectal pull through, sent to the Dept of Histopathology formed a part of this study from July 2012 till Sept 2013. There were 42 cases from which 73 samples were available. The surgical rectal biopsies were taken 2–3 cm above the pectinate line to avoid the physiological hypoganglionic zone. Samples during the colostomy closure were taken to assess the proximal end for the presence of ganglion cells. Biopsy samples from one stage transanal endorectal pull through were taken at 5 cm, 10–15 cms, 20–25 cms and 30cms from the anal verge. No suction biopsies were taken as this method is in the process of installation in our institute. All specimens were received fresh in 0.9 % saline soaked filter paper/gauze piece and subjected to frozen sections, stained with rapid H&E stain/toluidine blue stain and AChE histochemistry. The remnant was put in formalin and processed for paraffin embedded routine, H&E stained sections and this is considered the gold standard in the diagnosis of HD. Both positive (from known cases of Hirschsprung disease) and negative (from normal rectal mucosa) control tissues were stained with each batch of staining with AChE, to ensure its technical accuracy.

### Frozen section

All biopsies were taken fresh and frozen immediately and not snap frozen. A magnifying glass was used to identify the mucosal surface which is pale and the serosa which is relatively congested. Once the surface was identified, the biopsy was mounted on the cryostat chucks, exactly vertical to the surface of the mucosa and cut in serial 10 μ, thick sections and stained

### Rapid H&E stain

**Fig. 1:**
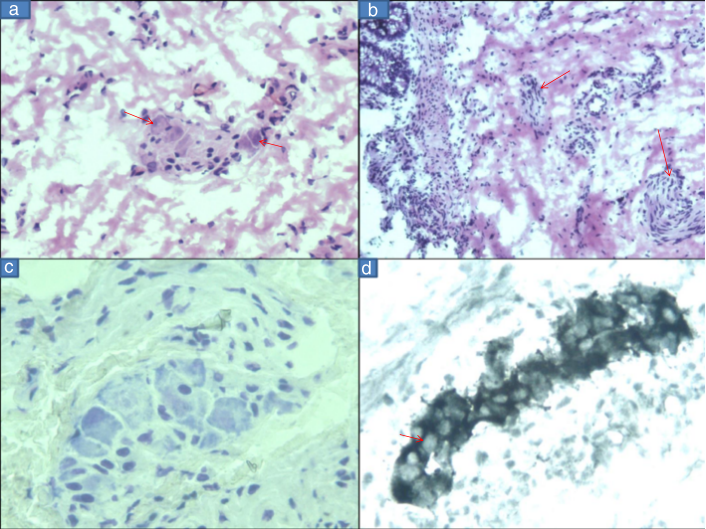
Frozen section stained with rapid H&E shows (**a**) The presence of GC (arrows) (**b**) Hypertrophied nerve trunks (arrows) in the SM and no GC (**c**) Toluidin blue stain shows the presence of GC. (**d**) Normal nerve plexus shows positive ie black staining in the cytoplasm with negative staining of the nuclei of GC which stand out as negative shadows (arrow)

Dip in freshly prepared Harris’ Hematoxylin for 15 s, rinse in tap water, dip in lithium carbonate or ammonia water, rinse in tap water, counter stain in eosin for 30–40 s, dehydrate with 5 quick dips in each of 2 changes of 95 % ethyl alcohol, 2 changes of absolute ethyl alcohol and 2 changes of xylene and mount. Sections were analyzed for orientation, adequacy, for the presence (Fig. 1a) or absence of ganglion cells and hypertrophied nerve trunks (Fig. 1b). If GC’s were absent, the block was exhausted, serial sections assessed for GC’s and if still not found the sample was labeled as ‘negative for GC’

### Toluidin blue stain

(Figure 1c)- Dip section in toluidin blue (1g in 100 ml distilled water) for 1 min, rinse in tap water, followed by 2 changes of 95 % ethyl alcohol, 2 changes of absolute ethyl alcohol and 2 changes of xylene. Mount wet.

### Rapid AChE staining protocol

In the present study, the rapid modified AChE staining technique of Kini et al. [6] was used. The original AChE staining method of Karnovsky and Roots [7] as detailed by Meier-Ruge [5] was further modified by Kini et al. [6] to suit a general histopathology laboratory in a developing country like India. The staining procedure takes 40 min.

### Interpretation of AChE histochemistry

The nerve fibers/RBC’s are stained black and serve as inbuilt controls. Cytoplasm of the GC stains positive (black) while its large nucleus is negative and appears as negative shadows in a black background (Fig. 1d). The patterns outlined are given below and are formulated by utilizing criteria similar to those initially described by Challa et al. [8].

### Pattern A

Nerve fibers in the muscularis mucosae, submucosa and in between the crypts in the lamina propria stain positive ie black. The nerve fibers travel in between the crypts and can reach the surface. The pattern resembles a tree. This is also known as the mature pattern as it is usually seen in infant’s > 3–6 months of age.

### Pattern B

Nerve fibers in the muscularis mucosae, submucosa and at the base of the crypts only stain positive ie black. No fibers travel up along the crypts. The pattern is similar to a tree pruned just above the trunk. This is also known as the immature pattern as it is usually seen in neonates and infants < 3 months.

### Equivocal pattern

Staining of nerve fibers occurs in the submucosa only. This pattern can be associated with or without hypertrophied nerve bundles in the submucosa, which is important to assess. Equivocal pattern with hypertrophied nerve trunks is suggestive of HD and corroborating with clinical scenario and radiological findings, a diagnosis of HD is offered. Equivocal pattern without hypertrophied nerve trunks is truly equivocal and a diagnosis of HD cannot be suggested. It is seen in cases of constipation due to other reasons.

### Negative pattern

No stained nerve fibers are seen in the muscularis mucosae and lamina propria, but small twigs are seen in the submucosa. The myenteric plexus or the submucosal GC however stains positive with negative shadows of the nuclei of the ganglion cells, which if present, strongly suggests the presence of ganglion cells and negates the diagnosis of HD. In a suction biopsy however this advantage is not there and a negative pattern may also indicate that the stain has not worked and demands a repeat.

A diagnosis of HD was made when no ganglion cells were seen on H&E/toluidin blue stained sections and a positive AChE pattern (A or B) was noted. Equivocal pattern with hypertrophied nerve trunks, suggested a diagnosis of HD.

## Results

In the 42 cases (73 samples) there were 33 males and 9 females. They were in the age range of one day to 11 years. There was one case of intestinal neuronal dysplasia (IND) which will be discussed separately, as the pathogenesis of this entity is different and comes under the broad umbrella of neurocristopathies. Final evaluation was done on 41 cases and 72 samples.

### Diagnosis on rapid H&E stain/toluidin blue

Frozen section rapid H&E/toluidin blue stain was done on 72 samples. Of these, 33 samples ie 46 % were negative for GC’s and 39 samples ie 54 % were positive for GC’s. Of the 33 samples negative for GC’s, 17 samples showed a positive AChE staining pattern and 19 samples showed absence of GC’s in routine paraffin embedded, H&E stained sections (after exhaustion of the block). Ten samples, which were small biopsies did not show the myenteric plexus due to orientation problem. Hypertrophied nerve trunks were present in 10 of the 33 samples which were negative for GC. The samples which were positive for GC (n-39) showed GC with ease in the myenteric plexus and at times in the submucosa. However, finding GC in the submucosa was more difficult because of the paucity, singly placed cells and widespread distribution. Yet, there were some cases where despite having given a diagnosis of ‘ganglion cells present’ we were unsure. This is because the neonatal GC’s are small and misleading. In rapid H&E and toluidine blue stain, an ideal GC appeared as a large cell with abundant cytoplasm and prominent nucleoli. However, the nucleoli were always better appreciated in toluidine blue stain making the pathologist more sure of a cell being a GC. Problems faced on frozen sections were due to orientation, small size of the biopsy and presence of a cell which was suggestive but not diagnostic of a GC. Cases in which we found cells suggestive, but not diagnostic of GC were considered as GC, if abundant cytoplasm was seen even if the nucleoli were not discernible.

### Diagnosis on AChE histochemistry

**Fig. 2:**
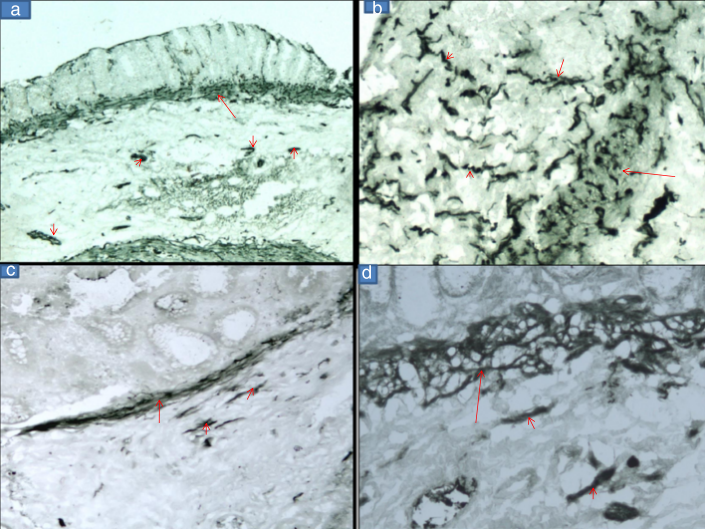
No 7791/13 c/o HD- AChE stain (**a**) Pattern A -At low power shows strongly positive nerve fibres in theMM (long arrow) and hypertrophied nerve trunks in the SM (small arrows) (**b**) Pattern A- MM showing strongly positive nerve fibres (long arrow)which are going up into the LP along the crypts (small arrows). No 19557/13 c/o HD AChE stain (**c**, **d**) Pattern B – Shows positively stained nerve fibres in the MM (long arrows) and SM (small arrows). No nerve fibres are seen in the LP

**Fig. 3:**
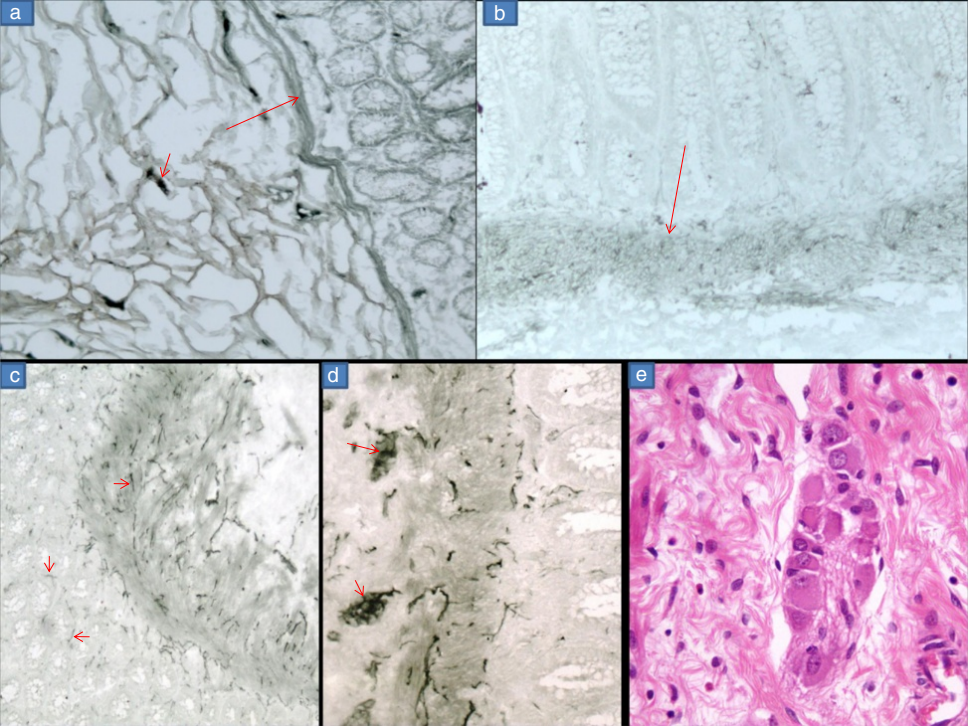
AChE stain –**a** & **b** shows negative stain ie no staining is seen in the MM (long arrow) and LP. Few small nerve twigs are positive in the SM (arrow). No 1265/13 c/o IND (**c**) AChE stain (pattern A) shows deep positive staining of the nerve fibres in the MM which are going up into the LP for a short distance (arrows) (**d**) AChE stain at high power showing two ganglia(arrows) in the SM showing negatively stained nuclei of the GC. **e** SM showing giant ganglia containing 14 GC’s H&E x 200

Of the 72 samples, 17 ie 24 % showed a positive pattern either A or B with AChE histochemistry. In all these 17 samples, no ganglion cell was identified on rapid H&E/toluidin blue stains and on paraffin embedded sections. Pattern A (Fig. 2a, b) was seen in 10 cases (9 were > 6 months of age and 1 was a day old child) and Pattern B (Fig. 2c, d) in 7 cases (2 were < 3 months of age and 5 were > 6 months) Hypertrophied nerve trunks stained strongly positive in 16 of the 17 cases. One case however showed a mixed pattern, which was predominantly pattern B, and hence was put in this category. Fifty five samples ie 76 % showed a negative pattern (Fig. 3a, b) of which 50 cases were totally negative and 5 samples showed an equivocal pattern without hypertrophied nerve trunks, which were however considered to be negative. On rapid H&E, 53 of these showed the presence of ganglion cells and 2 samples were negative for ganglion cells. These two negative samples, however belonged to 2 cases of total colonic aganglionosis (TCA) which normally shows a false negative pattern on AChE histochemistry. In approximately, two thirds of these cases, the myenteric plexuses were well discernable and showed a positive staining with AChE with clear cut negative shadows of the nuclei of the ganglion cells (Fig. 1d).

### Final analysis of 72 samples

On frozen section analysis with rapid H&E/toluidin blue stains, 33 samples were negative for ganglion cells, on AChE histochemistry, positive pattern staining was seen in 17 of these 33 samples and on paraffin embedded, H&E stained sections, 19 of these 33 samples showed absence of GC’s even after exhaustion of the block. Amongst these 33 samples, there were 2 samples of TCA which normally give a false negative staining with AChE histochemistry. So, after correction for the false negative stain, the number of samples showing a positive pattern with AChE histochemistry were 19 and all of these were negative for GC’s on paraffin embedded, H&E stained sections. These 19 samples belonged to 14 patients, Amongst them, there were 10 cases of classical HD and 2 cases each of long segment HD and total colonic aganglionosis.

### Sensitivity/specificity

Sensitivity and specificity of both tests ie frozen section rapid H&E/AChE histochemistry in the diagnosis of HD, were calculated taking paraffin embedded routine, H&E stained sections as the gold standard. Sensitivity of frozen sections rapid H&E in the diagnosis of HD is 57.57 % and specificity is 79.10 %. The p-value is <0.0001, which is statistically significant. The sensitivity of AChE histochemistry in the diagnosis of HD is 90.47 % and specificity is 96.36 %. The p-value is <0.0001, which is statistically significant.

### Intestinal neuronal dysplasia (IND)

There was one case of intestinal neuronal dysplasia, where the patient first came at the age of 13 months. He was clinically diagnosed as a case of HD, a colostomy was made and a rectal biopsy was taken, which was erroneously diagnosed as consistent with HD. This patient underwent Duhamel’s pull through surgery (ie colostomy closure) at the age of 2 years and the distal stump was sent for histopathology, which revealed the presence of increased ganglia and more than 20 % of submucosal ganglia were giant ganglia. The AChE stain showed a paradoxical (Pattern A) staining with increased staining of the fibers in the muscularis mucosae, which were going up for a short distance into the lamina propria (Fig. 3c). It also showed clusters of ganglion cells in the submucosa, showing negatively stained nuclei and deeply stained black cytoplasm(Fig. 3d). The original rectal biopsy was reevaluated and it showed the presence of GC’s and the serial sections also showed the presence of giant ganglia (Fig. 3e).

## Discussion

Hirschsprung disease (HD) is a developmental disorder characterized by the absence of enteric neurons/GC in the myenteric and submucosal plexuses (Meissners and Henles) of the distal parts of the gastrointestinal tract. It has an estimated incidence of 1 per 5,000 live births; however, this figure may be higher if perinatal demises are considered. Characteristic clinical presentation, is with large bowel obstruction which occurs at any time from birth to adulthood. Diagnostic modalities used are anorectal manometry, plain X-ray abdomen and barium enema.

Rectal biopsy (suction or surgical), taken 2–3 cms above the pectinate line, is the only way of making a definitive diagnosis and is mandatory before the definitive surgery. Paraffin embedded H&E stained section along with AChE histochemistry (done on part of the rectal biopsy which is kept frozen) as a double check, seems to be a perfect combination for making a conclusive diagnosis of HD, pre-operatively in the following scenario 1) Rectal biopsy taken from the correct site ie 2–3 cms above the pectinate line. Here, absence of ganglion cells and a positive AChE staining pattern clinches the diagnosis. 2) If the biopsy is taken from the normal physiological hypoganglionic area of the anorectal region, an erroneous diagnosis of HD will be made, as GC may not be found. Here a negative AChE stain will recommend a repeat biopsy from the proper site. 3) Rectal suction biopsy has now become the method of choice for the diagnosis of HD in most centers worldwide, wherein only the mucosa and the submucosa are present. The submucosal ganglion cell are fewer in numbers, are widely scattered, can be present singly and hence difficult to discern. Here, AChE staining pattern will be of great importance in making a diagnosis of HD 4) In neonates the GC’s are immature and hence are very difficult to be categorically labeled as a ganglion cells. Here again, a positive AChE staining pattern would help in diagnosis.

Intra-operative frozen section diagnosis for the presence or absence of GC, is needed in the following operative procedures 1) In instances where very sick children come with abdominal distension, HD is suspected but a colostomy has to be done as an emergency, without a preoperative diagnosis of HD. In such instances, intraoperative assessment of the ganglionic segment is done, four quadrant biopsies are taken from the doughnut to assess for ganglion cells by frozen section. 2) In colostomy closures, to assess for the presence of ganglion cells in the proximal segment that has to be anastomosed. 3) In cases of one stage endorectal pull through, where intraoperative biopsies are sent from 5 cm, 10–15 cms, 20–25 cms and 30 cms from the anal verge. Presence of conclusive ganglion cells at 30/20–25 cms, demarcates the proximal ganglionic segment, which is then anastomosed to the distal ganglionic bowel. 3) In total colonic aganglionosis for deciding upon the ganglionic segment for the colostomy site. Frozen section diagnosis have their limitations, the diagnosis of HD is given by the absence of ganglion cell i.e., it is a negative finding. The histopathologist has all the chance of missing that single ganglion cell, that may be present in the biopsy (false negative diagnosis) or labeling a cell as ganglion cell which actually may not be (false positive diagnosis), leading to a wrong diagnosis which is very catastrophic for the patient. Frozen sections have other problems like poor orientation and small size of the biopsy. Thus, we need ancillary techniques like AChE histochemistry, as a double check, for both diagnostic and in aiding the intraoperative procedures of HD. If still the intraoperative diagnostic opinion is equivocal, then the pathologist should ask the surgeon to perform the colostomy at a more proximal site and repeat biopsies should be sent from that site, for assessing the ganglion cells.

In the index study, 42 suspected cases of HD and 73 samples from them were evaluated and a final diagnosis of HD (10 cases of classical HD, 2 cases each of LSHD and TCA) was made in 14 cases and intestinal neuronal dysplasia in one case. The case of IND was removed from the final calculations as it does not come under the heading of HD, but under the broad umbrella of neurocristopathies. So, this study assessed 41 cases and 72 samples from them, for the presence of HD. Of the 14 cases of HD, there were 10 males (71.4 %) and 4 females (28.57 %) with a M: F ratio of 2.5:1. Schofield DE et al. [9] and Yang et al. [10] reported a M:F ratio of 4:1, 5.9:1 and 2.14:1 respectively. Hence, HD is more commonly seen in the males.

In the index study, on frozen section examination there were 33 samples negative for ganglion cells, which were reduced to 19 in the final result. Thus, in 14 samples the GC’s, despite being present, were not picked up on frozen section rapid H&E/Toluidin blue stains. This generally happens due to poor orientation of the biopsy, a very small biopsy that is usually sent, due to the immaturity of the ganglion cells and in suction biopsies, where only the submucosa is present, wherein the ganglion cells are usually sparse, singly scattered and small in size. Many of the above factors were operating in the index study as well. In this study the sensitivity of frozen sections in the diagnosis of HD is 57.57 % and specificity is 79.10 %. The p-value is <0.0001, which is significant. Rouzrokh et al. [11] evaluated 201 infants and children who underwent frozen section rectal biopsy to exclude HD. They found sensitivity on frozen section to be 85.8 % and specificity to be 90.2 %.

In the index study, on AChE histochemistry 17 samples showed a positive staining pattern (corresponding to absence of ganglion cells), but in the final result there were 19 samples which showed the absence of ganglion cells. Thus, there was false negative result in 2 cases, and both these cases were of total colonic aganglionosis. In them, a false negative staining is usually seen on AChE histochemistry and this pitfall is widely recognized. In the current study, the sensitivity of AChE histochemistry in the diagnosis of HD is 90.47 % and specificity is 96.36 % . The p-Value is <0.0001 which is significant. Thus, AChE histochemistry is an excellent ancillary technique and a double check in confirming the presence or absence of ganglion cells. Well et al. [12], Nakao et al. [13] and Park et al. [14] found a sensitivity/specificity of 64.90 %/98.70 %; 91 %/100 % and 92 %/100 % respectively. In the current study, of the 17 samples, Pattern A was seen in 10 samples and 9 of these were above 6 months of age and Pattern B was seen in 7 cases and only 2 were < 2 months. Hence, the Pattern B did not correlate well with age. Schofield et al. [9] in their study of 60 cases found Pattern A in 33 cases and Pattern B in 25 cases.

Although the use of AChE is of value, there are several potential problems, including false negative/positive results, the need for a frozen tissue, and the difficulty of reproducing the reaction in a reliable fashion. False positive staining [9, 15–17] is seen occasionally in hemorrhagic specimens (due to the high concentrations of AChE in the red blood cell membrane), in colitis and in intestinal neuronal dysplasia. False negative results [16, 18–20] occur in neonates, in TCA and in ultra short segment HD, technical factors, poor orientation of the minute biopsy, focal increase in AChE activity that may be missed and inexperienced hands. In the present study, false negative results were obtained in two cases of TCA and false positive staining was seen in a case of intestinal neuronal dysplasia. Hence, this technique is very good in picking up other neurocristopathies as well. If the biopsy shows the absence of ganglion cells and the AChE staining is also negative, then either the biopsy has been taken from the physiological hypoganglionic area of the anorectal region or it is a case of total colonic aganglionosis. If the AChE staining is positive and giant ganglia are seen, then IND should be ruled out.

There was one case of HD, redesignated as intestinal neuronal dysplasia, where the alert was sounded by AChE histochemistry. AChE stain revealed clusters of ganglion cells in the submucosa, showing negatively stained nuclei and deeply stained black cytoplasm along with pattern A staining with increased staining of the fibers in the muscularis mucosae, which were going up for a short distance into the lamina propria. IND shows a false positive pattern with AChE, which is well documented in literature [21]. This case met with the revised criteria of IND laid by Meier Ruge [22] in 2006–1) A minimum of 25 submucosal ganglia must be analyzed 2) More than 20 % of submucosal ganglia must be giant ganglia 3) A giant ganglia should contain >8 nerve cells per cross section. 4) Patient must be older than 1 year.

In the present study, the rapid modified AChE staining technique of Kini et al. [6] was used. The original AChE staining method of Karnovsky and Roots, modified many a times, was further modified by Kini et al. to suit a general histopathology laboratory in a developing country like India. This staining procedure takes 40 min. Due to complex technology of preparation and lack of pathologists’ experience, AChE histochemistry seems cumbersome and thus is used in specialized pathology centers only. But once the technique is mastered, there is no looking back. However, modern commercial diagnostic sets, using modified lyophilized media are in market and they will no doubt, increase the number of laboratories using AChE histochemistry for both preoperative diagnosis and in aiding the intraoperative procedures of HD.

Several immunohistochemical markers have been tried to look for ganglion cells in paraffin embedded tissues. Recently two new markers [23], calretinin and microtubule associated protein-2 (MAP-2) have been used as an adjunct in the diagnosis of HD, but these cannot be used in the intraoperative setting.

## Conclusion

Acetylcholinesterase (AChE) histochemistry is a very useful ancillary technique in the diagnosis and in aiding the operative procedures of HD. It acts as a double check in the diagnosis of HD.

## Abbreviations

AChE: acetylcholine esterase enzyme
GC: ganglion cell
HD: Hirschsprung’s disease
H&E: haematoxylin and eosin stain
IND: Intestinal neuronal dysplasia
LP: lamina propria
MM: muscularis mucosa
SM: Submucosa
TCA: total colonic aganglionosis
